# Gut microbiome–metabolome profiling reveals divergent growth performance in the spotted knifejaw (*Oplegnathus punctatus*)

**DOI:** 10.1038/s41598-026-50031-1

**Published:** 2026-04-24

**Authors:** Chao Ma, Chunxiu Chen, Xiaodi Shang, Yaoxin Wang, Houfu Liu, Yanguang Yu, Qunshan Wang, Xuehui Wang, Lei Jia, Shuang Liang

**Affiliations:** 1 Tianjin Fisheries Research Institute, Tianjin, 300221 China; 2 Tianjin Key Laboratory of Aqua-ecology and Aquaculture, College of Fisheries, Tianjin Agricultural University, Tianjin, 300384 China

**Keywords:** *Oplegnathus punctatus*, Gut microbiome, Metabolome, Growth performance, Recirculating aquaculture systems, Microbiology, Molecular biology

## Abstract

Growth divergence in recirculating aquaculture systems (RAS) compromises production efficiency, yet gut microbiome–metabolome correlations linked to this variation in the spotted knifejaw (*Oplegnathus punctatus*) remain unclear. To explore such associations, we analyzed intestinal microbiota and metabolites in pooled samples from fast-growing (FGC) and slow-growing (SGC) groups (*n* = 5 per group) after 6 months of RAS rearing. FGC showed significantly higher body weight (*P* < 0.05), lower microbial alpha diversity and beta dispersion, and was dominated by Proteobacteria (> 75%). SGC exhibited higher diversity and enrichment of Firmicutes, Bacteroidota, and Actinobacteriota. LEfSe (LDA score > 4.0) identified *Photobacterium* and *Cetobacterium* as taxa enriched in FGC, while *Lactobacillus*, *Weissella*, and *Pediococcus* were enriched in SGC. Metabolomic analysis identified 251 metabolites that were differentially abundant between FGC and SGC groups. In FGC, histamine, pyridoxine, and 6-hydroxyhexanoate showed significantly increased abundance, while microcystin LR and uracil showed decreased abundance (*P* < 0.05). KEGG pathway enrichment analysis indicated significant associations with protein digestion and absorption, ABC transporters, and aminoacyl-tRNA biosynthesis pathways. Correlation analysis revealed significant positive associations between FGC-enriched genera and metabolites potentially associated with growth performance. This study characterizes gut microbiome–metabolome correlation patterns associated with growth divergence in *O. punctatus*. Due to the small sample size and pooled sampling design, these findings represent exploratory observations that require validation in larger, individually-sampled cohorts before any functional interpretations or practical applications can be considered.

## Introduction

The spotted knifejaw (*Oplegnathus punctatus*) is an important mariculture economic fish in China, valued for its rapid growth, superior flesh quality, and strong disease resistance, it plays an important role in inshore and offshore mariculture^1,2^. However, during actual culture processes, individuals of the same batch of *O. punctatus* generally exhibit significant growth heterogeneity. This phenomenon leads directly to uneven marketable size, prolonged culture cycles, and diminished feed conversion efficiency, severely constraining improvements in industrial profitability^3^. Notably, growth heterogeneity is not unique to *O. punctatus*; it has been commonly reported in various other important economic fish species, such as large yellow croaker (*Larimichthys crocea*)^4^, marbled eel(*Anguilla marmorata*)^5^, striped catfish (*Pangasianodon hypophthalmus*)^6^, and grass carp (*Ctenopharyngodon idella*)^7^. and has emerged as a key constraint restricting the efficient development of the aquaculture industry, particularly hindering the standardized and intensive cultivation of *O. punctatus*.

Fish growth performance is a pivotal indicator for evaluating the economic efficiency of aquaculture systems. Fish growth is governed by a complex interplay of intrinsic and extrinsic factors, including genetics, nutrition, environment, and husbandry^8^. Recent studies have demonstrated that gut microbiome -host interactions s serve as a key regulator of physiological metabolism in animals. Fish intestinal microbes are capable of participating in and regulating a range of core physiological activities, such as nutrient digestion and absorption, energy metabolism and immune regulation, thereby exerting remarkable effects on host growth and development^9–11^. Existing research has demonstrated that disparities in growth performance are tightly linked to the composition and functional profiles of the gut microbiome. Fast-growing individuals of Anguilla marmorata harbor greater alpha-diversity in their gut microbial community, and the relative abundance of specific bacterial genera such as *Acinetobacter* exhibits a significant positive correlation with host growth rate^12^; In fast-growing populations of Cyprinus carpio, the intestinal levels of critical metabolites—such as spermidine, carnosine, and unsaturated fatty acids—are significantly elevated. Furthermore, the biosynthetic pathways responsible for these metabolites are tightly associated with particular microbial taxa (e.g., *Cetobacterium*)^13^; Dietary and waterborne probiotic supplementation significantly enhances growth performance in *Coilia nasus*, while simultaneously increasing intestinal levels of short-chain fatty acids (SCFAs) and bile acids^14^. Accumulating evidence has highlighted that the crosstalk between gut microbiome and host constitutes a critical component in regulating animal physiological metabolism. Fish gut microorganisms can participate in numerous essential physiological processes, including nutrient digestion and absorption, energy metabolism and immune regulation, thus imposing significant impacts on the growth and development of the host.

Metabolomics technology can systematically analyze dynamic changes of small-molecule metabolites in organisms, directly characterizing the functional output of gut microbiome -host interactions.In investigations of differential growth performance in fish, metabolomic technologies have identified significant disparities in key metabolic pathways—including amino acid metabolism, lipid metabolism, and energy metabolism—between fast-growing and slow-growing individuals^13,15–17^. Combining 16 S rRNA gene sequencing with untargeted metabolomics technology allows for the joint analysis of microbiome structural characteristics and functional metabolic profiles, thereby clarifying the molecular mechanism by which the gut microecosystem regulates host growth. This integrated research strategy has proven to be a powerful tool for investigating growth mechanisms in diverse farmed fish species^18,19^.

Currently, research related to *O. punctatus* mainly focuses on areas such as genetic breeding^20^, digestive system development^21^, immune gene function^22^, and economic evaluation of culture models^3^. However, whether the observed growth heterogeneity in *O. punctatus is* attributable to compositional and functional differences in the gut microbiome, or merely to environmental or genetic variation, remains unknown and requires systematic investigation.Therefore, this study integrates 16 S rRNA gene sequencing and untargeted metabolomics technology to systematically compare the gut microbiome structure and metabolic characteristics between fast-growing and slow-growing *O. punctatus* individuals. This study aims to identify key microbial taxa and differential metabolites significantly associated with growth heterogeneity, clarify the core metabolic pathways driving growth differentiation, and elucidate the molecular mechanism by which gut microbiome -metabolite interactions regulate host growth phenotypes. This study will clarify the microecological regulatory basis of growth heterogeneity in *O. punctatus*, and provide a theoretical foundation for developing targeted gut microbiome modulation strategies to improve the aquaculture efficiency of this species.

## Materials and methods

### Gut content sampling

Spotted knifejaw (*Oplegnathus punctatus*) were acquired from Tianjin Xingsheng Sea and Freshwater Aquaculture Co., Ltd. A total of 4,000 juveniles with an average body length of 6 cm and body weight of 6 g were cultured in a recirculating aquaculture system (RAS). Fish were fed a commercial marine fish compound feed (crude protein ≥ 48%, crude fat ≥ 10%) twice daily. Water quality parameters were stably maintained in the recirculating aquaculture system (RAS) during the experiment: water temperature 26 ± 0.5 ℃, salinity 25 ± 0.5, dissolved oxygen ≥ 6 mg/L, pH 7.6 ± 0.2, ammonia nitrogen ≤ 0.1 mg/L, nitrite nitrogen ≤ 0.05 mg/L.

After six months of rearing, 3,862 fish survived with a survival rate of 96.55%. Growth traits including body weight (BW), body length (BL), total length (TL), body height (BH) and body thickness (BT) were measured. Based on body weight distribution, the top 15 fish were assigned to the fast growth group (FGC) and the bottom 15 to the slow growth group (SGC), resulting in a total of 30 experimental fish. These fish were subsequently anesthetized with MS-222, after which their gut contents were aseptically collected using sterile surgical scalpels under strict aseptic conditions. Each group was further divided into five biological replicates, with each replicate consisting of pooled intestinal contents from three individual fish; the contents within each replicate were then mixed and homogenized thoroughly. All collected samples were immediately snap-frozen in liquid nitrogen and stored at −80 °C until subsequent metabolomics and intestinal microbial community analyses.It should be noted that the use of pooled samples (three individuals per replicate) and the limited number of biological replicates (*n* = 5 per group) constrain the statistical power of this study and preclude assessment of individual variation. Consequently, all findings should be interpreted as preliminary observations requiring validation in larger, individually-sampled cohorts.

All fish were subjected to identical feeding regimes and stable rearing conditions throughout the experiment to eliminate the interference of feeding and environmental factors on growth heterogeneity.

### Microbiome sequencing and analysis

Gut microbiome composition of 10 pooled samples (5 from FGC and 5 from SGC, derived from 30 fish in total) was determined by 16 S rRNA gene sequencing. Total genomic DNA was extracted from gut contents using the Mag-Bind Soil DNA Kit (Omega Bio-tek), and its concentration and purity were verified by 1% agarose gel electrophoresis, Qubit^®^ 2.0 Fluorometer (Thermo Scientific), and Agilent Bioanalyzer 2100 system (Agilent).

The V3–V4 hypervariable region of the 16 S rRNA gene was amplified with barcoded specific primers 341 F (5′-CCTAYGGGRBGCASCAG-3′) and 806R (5′-GGACTACNNGGGTATCTAAT-3′) using a Bio-Rad T100 PCR instrument (Bio-Rad). PCR reactions were performed in 30 µL volumes containing 15 µL 2× Phusion^®^ High-Fidelity PCR Master Mix (New England Biolabs), 0.2 µM of each primer, and ~ 10 ng template DNA. Thermal cycling conditions: 98 ℃ for 1 min, followed by 30 cycles of 98 ℃ for 10 s, 50 ℃ for 30 s, 72 ℃ for 30 s, and a final extension at 72 ℃ for 5 min.

PCR products were verified by 2% agarose gel electrophoresis, and samples with a 400–450 bp main band were selected, pooled in equidensity ratios, and purified using the Qiagen Gel Extraction Kit (Qiagen) for library construction. High-throughput sequencing was conducted by Shanghai Applied Protein Technology Co., Ltd. on the Illumina NovaSeq6000 PE250 platform (Illumina, USA).

Raw reads were first assigned to individual samples based on barcodes, then paired-end reads were merged using FLASH to generate consensus sequences. Clean reads were imported into QIIME2, where DADA2 was used for denoising, quality filtering, and Amplicon Sequence Variant (ASV) inference (100% sequence similarity). Taxonomic annotation was performed against the SILVA 138 database.

Alpha diversity indices (Chao1, Observed Species, Shannon, Simpson) and rarefaction curves were generated using QIIME2 to assess within-sample diversity. Microbial community composition at the phylum and genus levels was visualized using bar plots and heatmaps.

Beta diversity was analyzed via PCoA (based on Unweighted UniFrac distance) and NMDS (based on Bray-Curtis distance), with ANOSIM and adonis tests used to validate the statistical significance of inter-group differences. Differentially abundant taxa were identified by STAMP (Wilcoxon rank-sum test, *P* < 0.05). LEfSe analysis was applied to identify taxa with significant differential abundance between groups (LDA score > 4.0), focusing on core differential taxa for subsequent correlation analysis.

### LC/MS untargeted metabolomic analysis

Untargeted metabolomics analysis of intestinal metabolites was performed on 10 samples (*n* = 5 per group) by Shanghai Applied Protein Technology Co., Ltd. (Shanghai, China). Immediately after collection, intestinal samples were snap-frozen in liquid nitrogen and stored at − 80 °C until analysis. For metabolite extraction, ~ 80 mg of frozen intestinal tissue was transferred to 2 mL Eppendorf tubes containing 200 µL ultrapure H₂O and five ceramic beads on dry ice. Samples were homogenized, followed by the addition of 800 µL methanol/acetonitrile (1:1, v/v). The mixture was centrifuged at 14,000 × g, 4 °C for 20 min. The supernatant was dried in a vacuum centrifuge, re-dissolved in 100 µL acetonitrile/water (1:1, v/v), and centrifuged again at 14,000 × g, 4 °C for 15 min. The final supernatant was injected for ultra-high-performance liquid chromatography quadrupole time-of-flight mass spectrometry (UHPLC-Q-TOF MS) analysis.

QC samples, prepared by pooling aliquots of all supernatants, were analyzed to monitor instrument and analytical stability; features with a relative standard deviation (RSD) > 30% were discarded. Raw data were converted to MzXML format using ProteoWizard MSConvert, processed with XCMS for peak detection and CAMERA for annotation, and metabolites were identified by matching against an in-house standard database. Multivariate analyses (PCA, OPLS-DA) and differential metabolite screening (VIP > 1, *P* < 0.05, fold change > 1 or < 1) were performed using the R package ropls.

Differential metabolites were identified and annotated, then mapped to corresponding biochemical pathways via metabolic enrichment analysis based on the KEGG database (https://www.genome.jp/kegg/)^23,24^. A butterfly plot was constructed using the ggplot2 R package to visualize the log₂ fold change (log₂FC) values of the top 10 most statistically significant elevated and decreased metabolites.

### Statistical analysis

Data comparisons between FGC and SGC were performed using SPSS 27.0 software. Student’s t-test was used for normally distributed data, and the Mann-Whitney U-test was applied for non-normally distributed data. Differences were considered statistically significant at *P* < 0.05. Data were expressed as mean ± SEM.

Spearman correlation coefficients were calculated to assess relationships among significantly differential metabolites. Hierarchical clustering analysis based on Spearman correlations between differential gut microbiota and metabolites was performed using the heatmap package in R (Version 3.4.2), with correlation coefficients (r) ranging from − 1 to + 1.

## Results

### Growth performance of *Oplegnathus punctatus*

Following a 6-month rearing period in a recirculating aquaculture system, 30 *Oplegnathus punctatus* specimens exhibited body weights ranging from 89.90 to 355.10 g. Individuals weighing 89.90–147.10 g (50.0% of total) were classified as the slow-growing control SGC, while those in the 177.47–355.10 g range (remaining 50.0%) formed the fast-growing control FGC for subsequent analyses of growth performance, gut microbiome, and metabolomic profiles.

Significant intergroups differences in growth metrics were observed (Table 1). The FGC demonstrated significantly greater values in body weight (248.74 ± 55.41 g vs. 123.22 ± 18.91 g, *P* < 0.001), body length (17.61 ± 1.05 cm vs. 14.60 ± 1.53 cm, *P* < 0.001), and total length (21.93 ± 1.64 cm vs. 17.43 ± 0.96 cm, *P* < 0.001) compared to the SGC. Statistically significant differences were also noted in body height (9.72 ± 0.94 cm vs. 8.24 ± 0.37 cm, *P* = 0.001) and body thickness (2.08 ± 0.40 cm vs. 1.58 ± 0.17 cm, *P* = 0.006). In summary, the growth performance of *Oplegnathus punctatus* in the FGC is significantly better than that in the SGC.

**Table 1:** Growth performance of *Oplegnathus punctatus* in the FG and SG groups.

items	FGC	SGC	*P*
BW(g)	248.74 ± 55.41	123.22 ± 18.91	0.000
BL(cm)	17.61 ± 1.05	14.60 ± 1.53	0.000
TL(cm)	21.93 ± 1.64	17.43 ± 0.97	0.000
BH(cm)	9.73 ± 0.94	8.24 ± 0.36	0.001
BT(cm)	2.08 ± 0.40	1.58 ± 0.17	0.006

BW, body weight; BL, body length; TL, total length; BH, body height; BT, body thickness.

### Community structures of gut microbiome

The gut microbiome of the FGC and SGC was analyzed by 16 S rRNA gene sequencing on the Illumina NovaSeq6000 PE250 platform (Illumina, USA). After quality filtering, a total of 984,859 high-quality, non-chimeric clean reads were obtained from the 10 samples, with each sample yielding 86,656–106,944 reads. These reads were inferred as 3,092 Amplicon Sequence Variants (ASVs) at 100% sequence similarity via DADA2.To evaluate alpha diversity differences between groups, we analyzed Ace, Chao1, Shannon, and Simpson indices (Table 2). The SGC exhibited significantly higher microbial richness and diversity than the FGC (*P* < 0.05).

**Table 2:** Alpha-diversity indices of the gut microbiome of *Oplegnathus punctatus*.

Group	Richness estimator		Diversity index	
	Ace	Chao1	Shannon	Simpson
FGC	116.70 ± 29.26^a^	116.38 ± 29.05^a^	2.78 ± 0.11^a^	0.71 ± 0.02^a^
SGC	412.51 ± 102.50^b^	414.83 ± 102.05^b^	6.41 ± 1.55^b^	0.96 ± 0.02^b^

Data are the mean ± (SE). The different letters behind the data indicate a significant difference (*P* < 0.05) between the two groups.

**Fig. 1:**
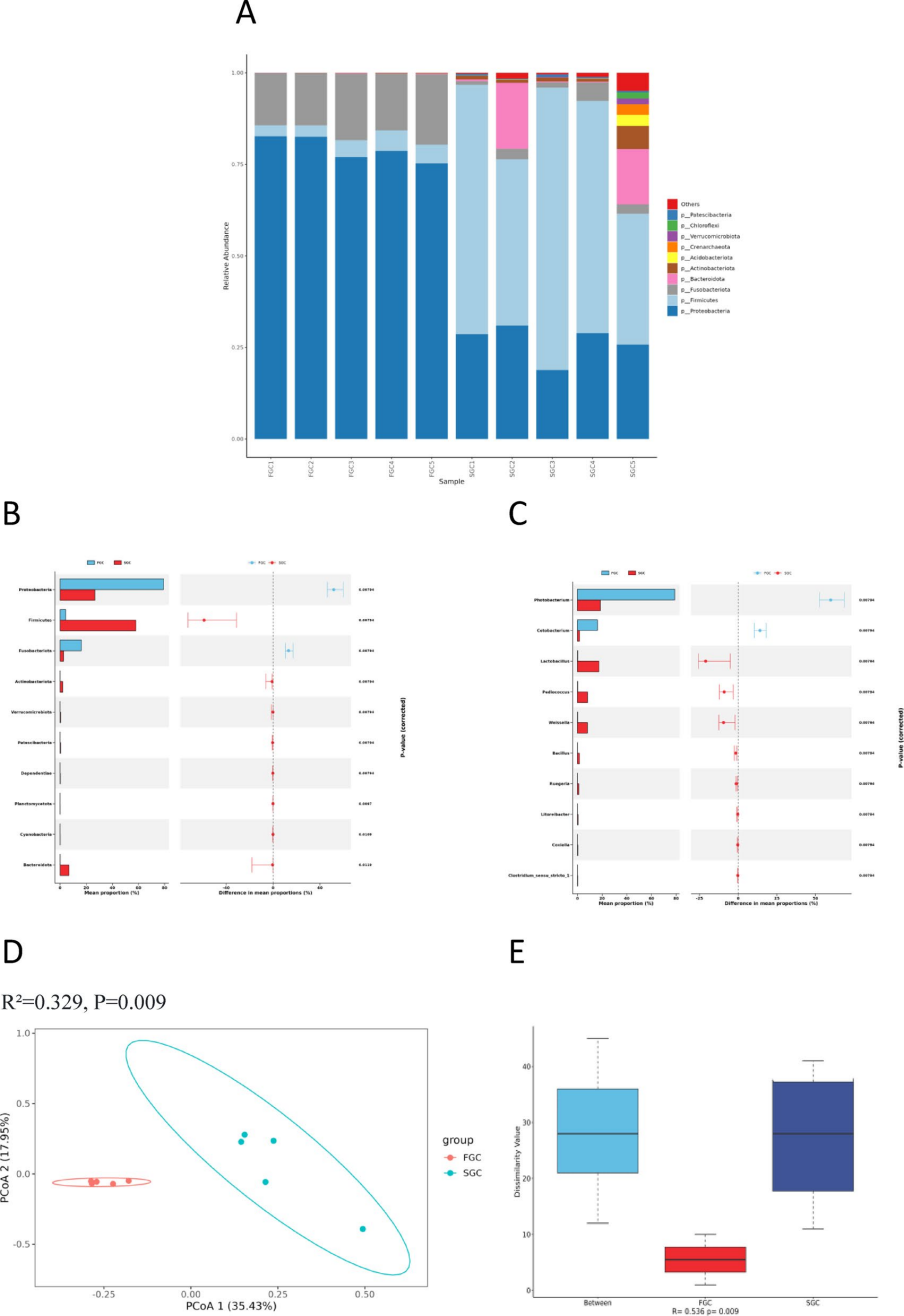
Community analysis of gut microbiome of *Oplegnathus punctatus* from the FGC and SGC. (**A**) The relative abundances of gut microbiome at phylum level; (**B**) STAMP analysis (Wilcoxon rank-sum test) of gut microbiome at the phylum levels; (**C**) STAMP analysis (Wilcoxon rank-sum test) of gut microbiome at the genus level; (**D**) PCoA based on Unweighted UniFrac distance revealed distinct separation between FGC and SGC, and PERMANOVA (Adonis) confirmed extremely significant differences (R²=0.329, *P* = 0.009); (**E**) ANOSIM analysis based on Unweighted UniFrac distance (*R* = 0.536, *P* = 0.009).

Analysis of the phylum-level relative abundance (Fig. 1A) demonstrated that the gut microbiome of the FGC was dominated by Proteobacteria, with its relative abundance exceeding 75% across all FGC samples. Conversely, the relative abundance of Proteobacteria in the SGC decreased dramatically, while Firmicutes, Bacteroidota, and other taxa increased significantly, reflecting a fundamental difference in phylum-level microbiome composition between the two groups. STAMP analysis using the Wilcoxon rank-sum test showed that Proteobacteria and Fusobacteriota were significantly enriched in the FGC (*P* < 0.05), while Firmicutes, Actinobacteriota, and Bacteroidota were significantly more abundant in the SGC (*P* < 0.05) (Fig. 1B). At the genus level (Fig. 1C), *Photobacterium* and *Cetobacterium* were significantly enriched in the FGC *(P* < 0.05), whereas *Lactobacillus*,* Pediococcus*, and *Weissella* showed significantly higher abundance in the SGC (*P* < 0.05). PCoA analysis based on Unweighted UniFrac distance showed a distinct separation between the FGC and SGC. PERMANOVA (Adonis) test further verified extremely significant differences in gut microbial community structure between the two groups (R²=0.329, *P* = 0.009) (Fig. 1D). FGC samples were highly clustered, while SGC samples were more dispersed, indicating significant inter-group differences in gut microbial community structure. ANOSIM further confirmed the significant separation between the two groups (*R* = 0.536, *P* = 0.009; Fig. 1E).

**Fig. 2:**
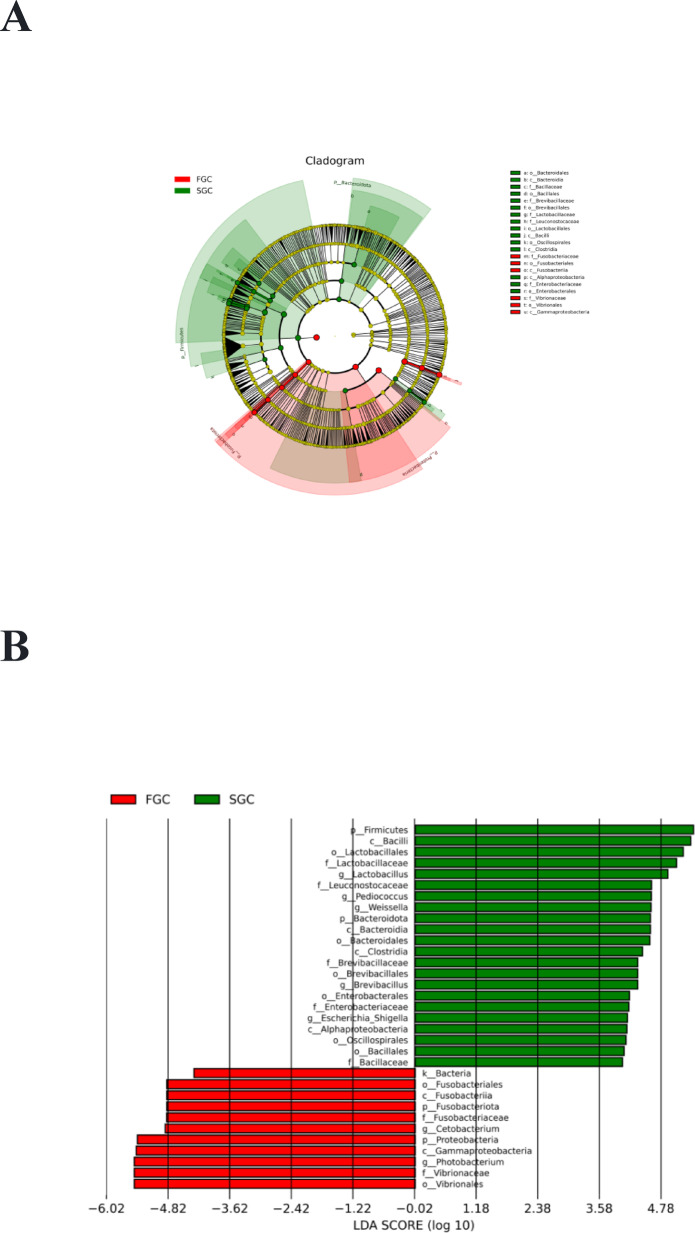
LEfSe analysis results. (**A**) CCladogram showing the hierarchical distribution of discriminant microbial taxa; (**B**) LDA effect size plot showing the LDA scores of significantly different taxa (LDA score > 4.0).

LEfSe analysis identified taxa with significant differential abundance between the two groups(Fig. 2A, B). For the FGC (red bars), multiple taxa with strong discriminatory power were significantly enriched, including the phylum Fusobacteriota, orders Vibrionales and Fusobacteriales, family Vibrionaceae, and *Photobacterium* (LDA = 5.47) and *Cetobacterium* (LDA = 4.87). In contrast, the SGC (green bars, LDA score > 4.78) was characterized by enrichment of the phyla Firmicutes and Bacteroidota, orders Lactobacillales, Bacteroidales, and Clostridiales, families Lactobacillaceae and Bacteroidaceae, and *Lactobacillus* (LDA = 4.92), *Weissella* (LDA = 4.59), and *Pediococcus* (LDA = 4.60). The cladogram (Fig. 2A) visualized the hierarchical taxonomic distribution of these differentially abundant taxa, while the LDA effect size plot (Fig. 2B) quantified their contribution to group differentiation, indicating that these taxa contribute substantially to the differentiation between fast- and slow-growing *O. punctatus.*

### Profiles of gut metabolites and KEGG pathways

**Fig. 3:**
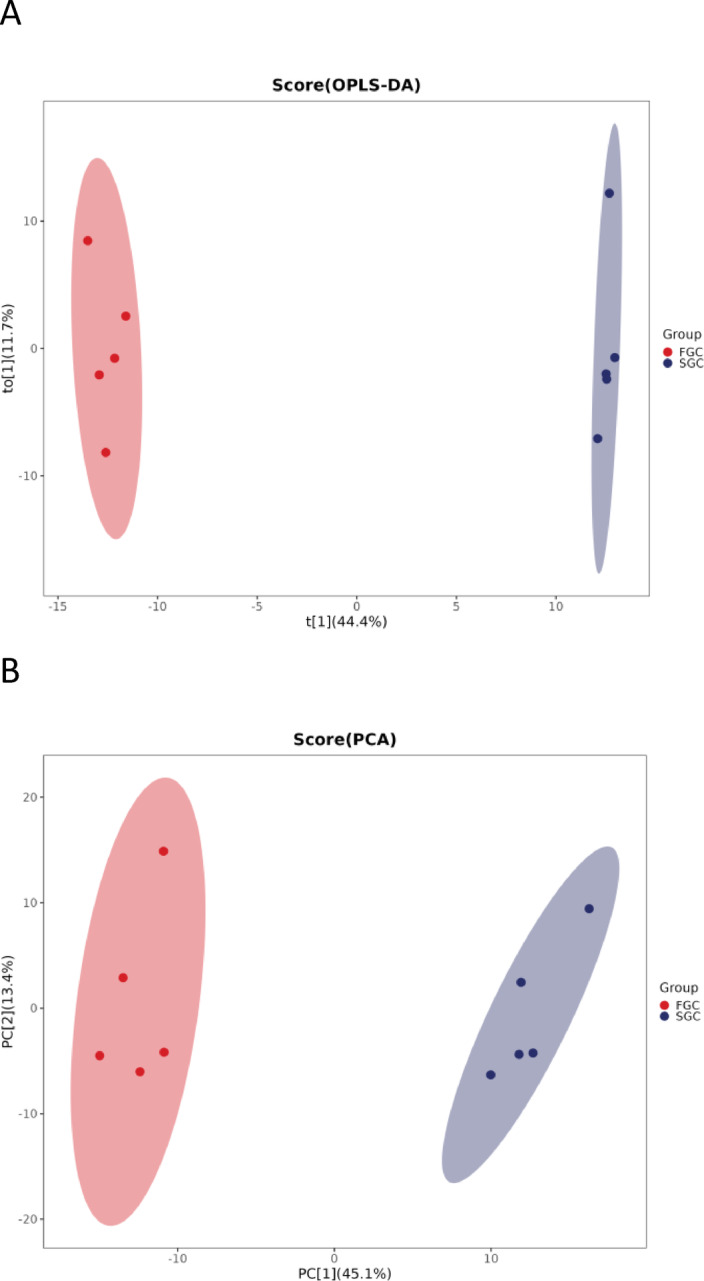

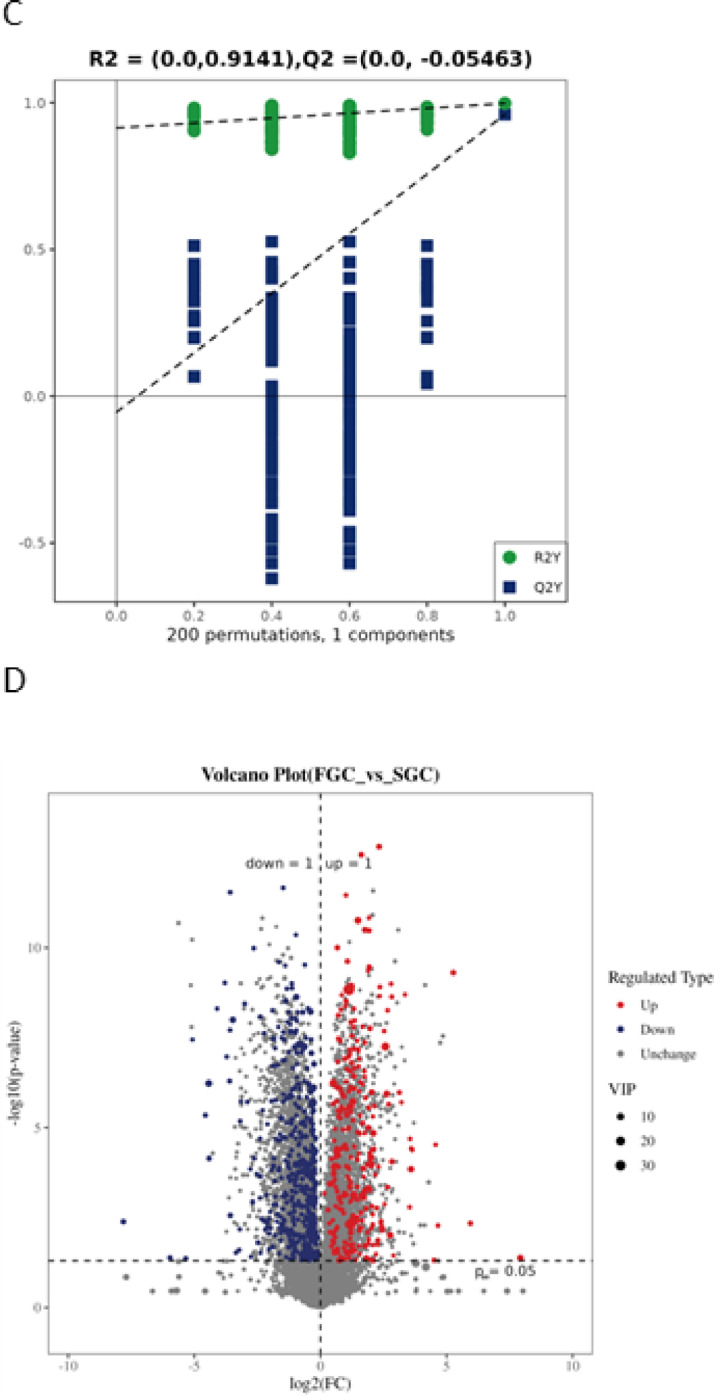

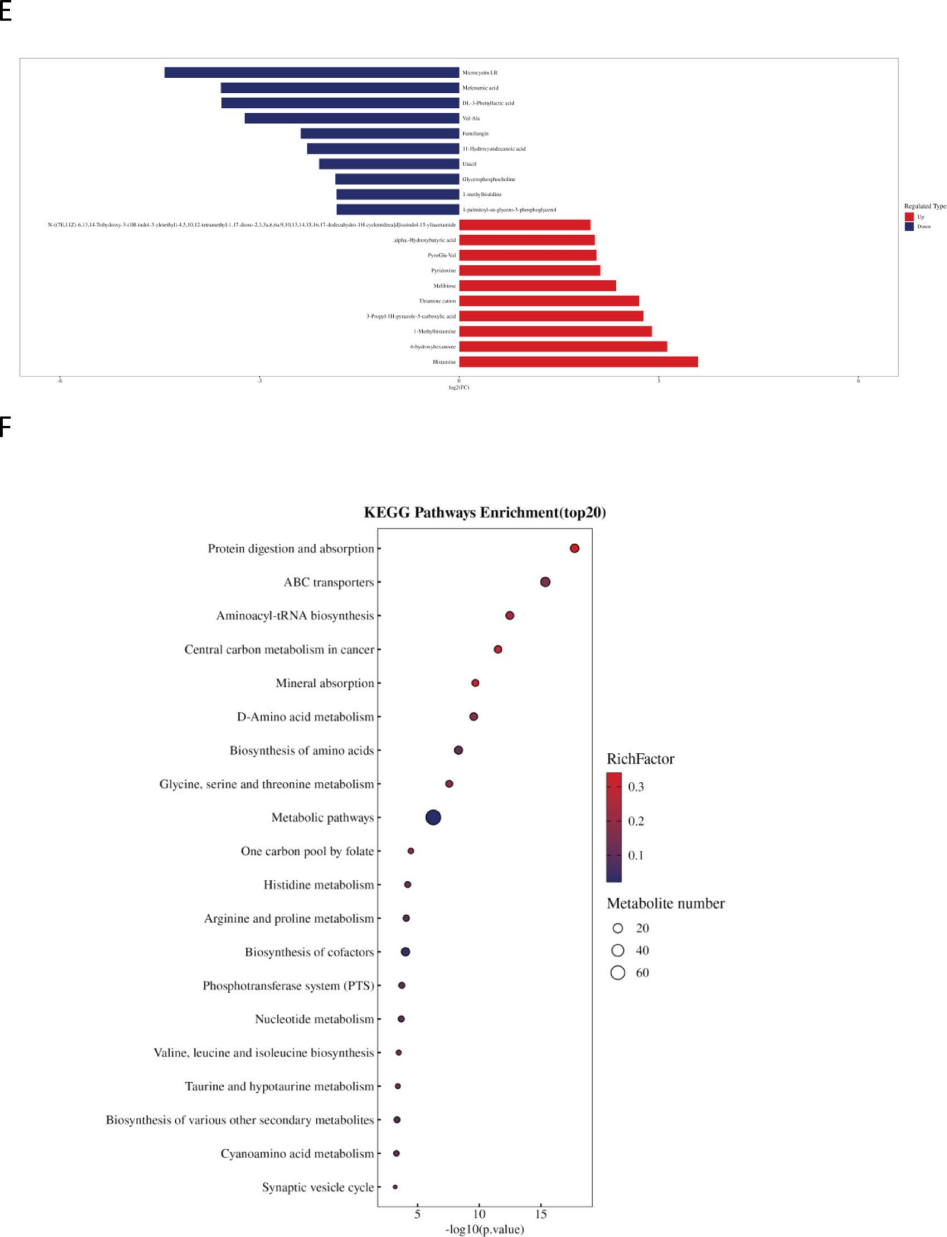
Characteristics of gut metabolites and KEGG pathways between the FGC and SGC groups of *Oplegnathus punctatus*. (**A**) PCA analysis of metabolites. The distance between coordinate points indicates the degree of aggregation and dispersion among samples, with red dots representing the FGC group and blue dots representing the SGC group; (**B**) OPLS-DA score plot; (**C**) Validation plot for the OPLS-DA model. The horizontal axis represents the permutation retention rate (200 permutations performed), and the vertical axis represents the permuted values of R2Y (green dots) and Q2Y (dark blue bars). The two dashed lines denote the regression lines of R2Y and Q2Y, respectively, confirming that the original model has no overfitting; (**D**) Volcanic map of differential metabolites. Red represents the upregulation; blue represents the downregulation; gray represents no significant change.(FC > 1/<1, *P* < 0.05, VIP > 1). (**E**) The butterfly plot.The top 10 most significantly altered metabolites between FGC and SGC groups. Metabolites with positive log₂FC values (red bars) are upregulated in the FGC, while those with negative values (blue bars) are downregulated. (**F**) KEGG pathway enrichment.The bubble chart depicts the top 20 significantly enriched KEGG pathways. The y-axis lists the pathway names, and the x-axis represents the statistical significance of enrichment (-log₁₀(p value)). The size of each bubble corresponds to the number of differential metabolites mapped to that pathway. The color of the bubbles indicates the enrichment factor (Rich Factor), with red representing a higher degree of enrichment.

We conducted untargeted metabolomics analysis on the intestinal samples of *Oplegnathus punctatus* with divergent growth rates. Both unsupervised principal component analysis (PCA, Fig. 3A) and supervised orthogonal partial least squares-discriminant analysis (OPLS-DA, Fig. 3B) revealed a distinct separation between the two groups, indicating significant differences in intestinal metabolite profiles between the FGC and SGC. The validity of the OPLS-DA model were further confirmed by permutation tests involving 200 permutation experiments.R2Y and Q2 of OPLS-DA model were 0.998 and 0.96 respectively, showing a good predictive ability (Fig. 3C).A total of 704 metabolites were detected in both positive ions and negative ions modes. Further analysis revealed 251 named differential metabolites, comprising 95 metabolites with increased abundance and 156 metabolites with decreased abundance (fold change > 1 or < 1, *P* < 0.05, VIP > 1) (Fig. 3D).

A butterfly plot was constructed to visualize the log₂ fold change (log₂FC) of the top 10 significantly increased and decreased metabolites (Fig. 3E). Histamine showed the most pronounced elevation in the FGC group, followed by 6-hydroxyhexanoate, 1-methylhistamine, 3-propyl-1 H-pyrazole-5-carboxylic acid, thiamine cation, and pyridoxine. Conversely, microcystin LR exhibited the strongest reduction, accompanied by significant decreases in mefenamic acid, DL-3-phenyllactic acid, Val-Ala, and fumifungin.

To elucidate the functional implications of differential metabolites, KEGG pathway enrichment analysis was performed. Among the top 20 significantly enriched pathways (Fig. 3F), Protein digestion and absorption, ABC transporters, and aminoacyl-tRNA biosynthesis exhibited the highest statistical significance.

### Correlations between gut microbiome and metabolome

**Fig. 4:**
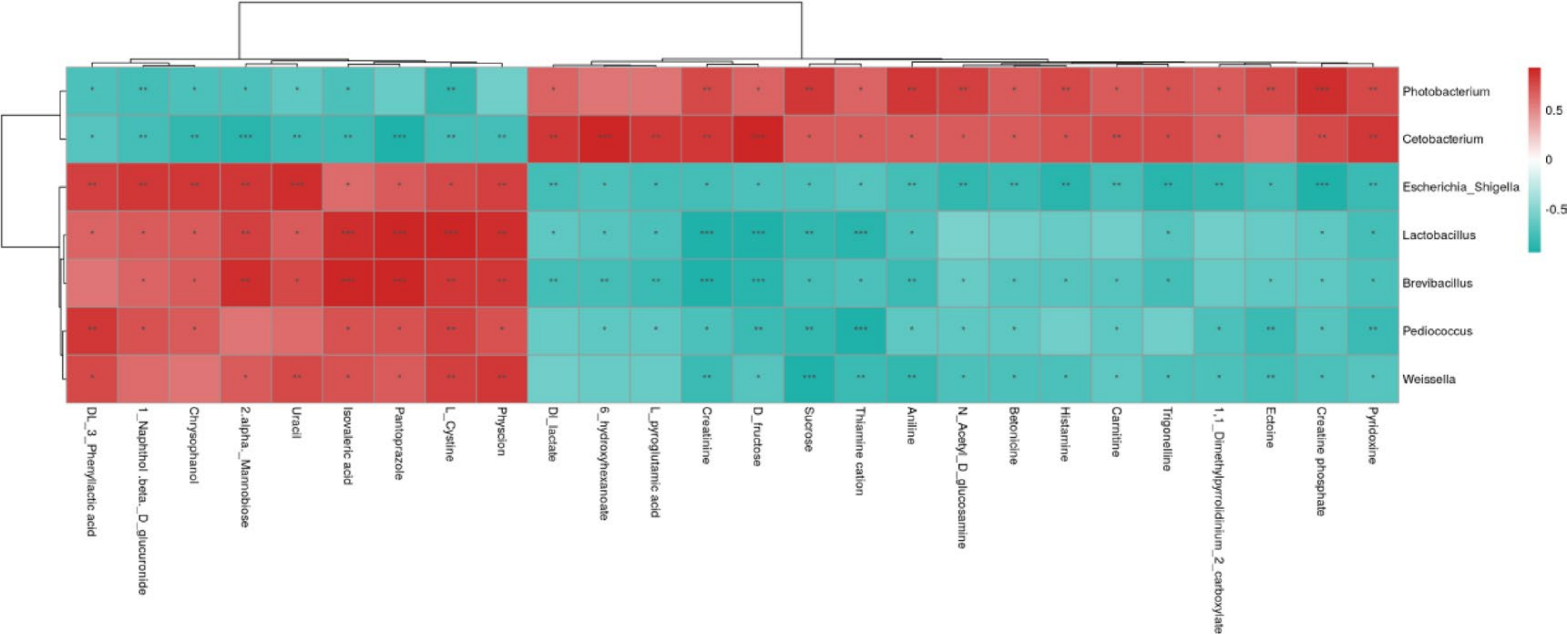
Correlation heatmap of differential gut microbiota and differential metabolites in *Oplegnathus punctatus* with different growth performance(Microbiota were selected with LDA > 4, and metabolites were selected with VIP > 1.5, *P* < 0.05, and FC > 2 or < 0.5. Red indicates positive correlations, while green indicates negative correlations. p-value < 0.05, indicated by *; p-value < 0.01, indicated by **; p-value < 0.001, indicated by ***.).

Correlation analysis was performed to explore the potential associations between differential gut microbiota (LDA score > 4) and differential metabolites (VIP > 1.5, *P* < 0.05, FC > 2 or FC < 0.5), aiming to clarify the interplay between gut microbes and metabolites. As shown in Fig. 4, *Cetobacterium* and *Photobacterium* were positively correlated with 17 metabolites. Specifically,*Cetobacterium* exhibited extremely significantly positive correlations with D-fructose and 6-hydroxyhexanoate (***P < 0.001),and both genera showed significantly positive correlations with pyridoxine and creatinine (**P < 0.01), together with other main positive metabolites including creatine phosphate and carnitine. Meanwhile, both genera were negatively correlated with 9 metabolites, such as L-cystine, uracil and isovaleric acid.

In contrast, *Escherichia-Shigella*,* Lactobacillus*,* Brevibacillus*,* Pediococcus and Weissella* displayed an opposite correlation pattern: they were negatively correlated with all 17 metabolites mentioned above, and positively correlated with the 9 metabolites that were negatively correlated with *Cetobacterium* and *Photobacterium*.

## Discussion

The gut microbiome may play a potential role in influencing fish growth and metabolic homeostasis, and its community structure is likely influenced by multiple factors, including host genetic background, diet formulation, and aquaculture environmental parameters^9,10,25,26^.In the present study, 16 S rRNA gene sequencing was performed to compare the gut microbial profiles between the FGC and SGC of *O. punctatus*. Our results showed that Proteobacteria was the dominant phylum in the FGC, a community characteristic that is consistent with earlier reports on the gut microbiome of various marine fish cultured in RAS^27,28^, where Proteobacteria generally comprise the most abundant component of the gut microbiome.Conversely, the relative abundance of Proteobacteria was significantly decreased in the SGC, whereas those of Firmicutes, Bacteroidota, and Actinobacteriota were significantly increased. These phyla have been detected to be significantly enriched in fish exhibiting slower growth rates under aquaculture conditions, which may reflect distinct host-microbe association states. These compositional shifts represent distinct microbial community patterns that have been correlated with variations in host growth performance in prior studies^13,29,30^. It should be emphasized that these compositional variations are only correlatively linked to growth phenotypes in the present study. Causal inferences regarding microbial effects on growth performance remain unsupported owing to pooled sampling and the limited sample size.

Alpha diversity analysis revealed that the SGC exhibited significantly higher Chao1, Shannon, and Simpson indices compared with the FGC. However, numerous studies have shown that the association between gut microbial alpha diversity and host health or growth performance is not a simple positive correlation under specific disease states or differential growth conditions. For instance, in diseased Yunlong grouper (*Epinephelus moara* ♀ × *Epinephelus lanceolatus* ♂), gut microbial diversity and richness remained unchanged, whereas notable shifts in the relative abundance of specific phyla were detected^31^.Similarly, investigations across different fish species with differential growth rates—including the large yellow croaker (*Larimichthys crocea*)^4^, common carp *(Cyprinus carpio*)^13^, and *Anguilla marmorata*^12^—consistently showed that fast-growing individuals may exhibit reduced alpha diversity relative to their slower-growing counterparts. Principal coordinates analysis (PCoA) based on unweighted UniFrac distances revealed significant separation between the two groups, with FGC samples forming tight clusters, suggesting a relatively consistent core microbiome structure. Given the pooled sampling design and small sample size, the statistical power to validate these diversity and structural patterns remains limited, and such observations should be regarded as cohort-specific rather than broadly generalizable. Collectively, these observations reflect distinct gut microbial community patterns between fast- and slow-growing *O. punctatus* in the present study.

LEfSe analysis (LDA > 4.0) identified *Photobacterium* and *Cetobacterium* as significantly enriched taxa in the FGC. *Photobacterium* represents a common genus in fish intestinal tracts and has been associated with chitin digestion in previous studies^10^; *Cetobacterium* is commonly found in marine fish cultured in RAS and has been correlated with various metabolic processes in prior research^30,32^.In our study, both genera were significantly enriched in FGC individuals, suggesting potential associations with growth performance that warrant further investigation. Conversely, the SGC exhibited significant enrichment of *Lactobacillus*,* Weissella*,* and Pediococcus.* Although these genera are frequently applied as exogenous probiotics to support aquatic animal health^33,34^, their increased abundance in the SGC was not associated with enhanced growth rates. Instead, this shift may reflect microbial niche adaptation corresponding to to altered host growth conditions. Studies suggest that differences in host growth could be associated with relative accumulation of undigested carbohydrates and other substrates in the intestinal lumen, which may create selective microhabitats that favor proliferation of acid-producing bacteria like lactobacilli^13,35^. Collectively, the FGC exhibited a Proteobacteria-dominated gut microbiome, while the SGC displayed higher microbial diversity with lactobacilli enrichment, showing distinct microbial community patterns correlated with growth phenotypes. These observations require validation through future studies with individual sampling and functional assays.

This study was performed to characterize intestinal metabolite profiles between FGC and SGC of *O. punctatus* using untargeted metabolomics. The FGC exhibited significantly elevated levels of histamine, pyridoxine, and 6-hydroxyhexanoate. Histamine is a key mediator of fish intestinal immunity as reported in previous studies^36,37^, which have also described its association with intestinal digestive enzyme activity in fish^38,39^.Pyridoxine, an essential water-soluble vitamin^40^, and prior research has linked it to aminoacyl-tRNA biosynthesis, amino acid metabolism, and protein metabolism related to fish growth performance^41^. 6-hydroxyhexanoate, a critical intermediate in both ω- and β-oxidation pathways of fatty acids, contributes to multiple metabolic processes by supplying energy for cellular proliferation and protein synthesis^42^.In contrast, MC-LR levels were significantly reduced in the FGC. As a toxic metabolite of cyanobacterial blooms, high concentrations of MC-LR have been reported to induce oxidative damage to the fish liver and disrupt core metabolic pathways including the TCA cycle and aminoacyl-tRNA biosynthesis^43^. Similarly, uracil levels were significantly decreased in the FGC; as a product of nucleic acid degradation, this change has been hypothesized to reflect altered energy allocation toward growth-related metabolic processes in previous work^42^.These differential metabolites were significantly enriched in pathways related to protein digestion/absorption, ABC transporters, and aminoacyl-tRNA biosynthesis (*P* < 0.05). These findings align with previous reports in sea bass *(Lateolabrax maculatus*)^42^, large yellow croaker *(Larimichthys crocea*)^44^, Atlantic salmon (*Salmo salar*)^45^. The enrichment of ABC transporter pathways has also been associated with transmembrane transport activity for amino acids, oligopeptides, and vitamins in the fish intestine in prior studies^46^. These metabolomic findings are consistent with the gut microbiome profiling results, collectively showing correlative patterns between intestinal metabolite profiles and growth phenotypes in this exploratory study.

Spearman correlation analysis was performed to explore potential associations between differentially abundant microbial genera (LDA score > 4.0) and differential metabolites, and a preliminary correlation network between gut microbiota and host metabolites was constructed. Our analysis showed that FGC-enriched genera (*Photobacterium*,* Cetobacterium*) exhibited positive correlations with metabolites significantly elevated in the FGC, including 6-hydroxyhexanoate, histamine, and pyridoxine, while showing negative correlations with SGC-enriched metabolites such as uracil, isovaleric acid, and L-cystine. Previous studies have reported that Cetobacterium is involved in vitamin B12 biosynthesis^47^, and can produce extracellular enzymes that hydrolyze complex carbohydrates^17^, which may be linked to the elevated levels of B vitamins and energy-related metabolites observed in the FGC in this study. This may partly explain the elevated levels of B vitamins and energy-related metabolites in the FGC, further supporting the improved nutritional metabolism and growth performance of *O. punctatus.* Pyridoxine, as a key cofactor in enzymatic reactions, has been widely reported to be associated with the metabolism of amino acids, lipids, and carbohydrates in previous work^40,42^. Conversely, although lactobacilli enrichment has been associated with immunomodulatory effects in fish in prior studies^48^, their proliferation in SGC likely represents a passive response to intestinal substrate accumulation under reduced host metabolic activity. Multi-omics association analysis collectively indicated that the coordinated microbiome-metabolite patterns were significantly correlated with growth phenotypes in *O. punctatus*. These findings are consistent with comparable studies in multiple marine fish species^26,27,49^, which have also reported associations between intestinal microecosystems and host growth phenotypes^35^.Owing to the constraints of pooled sampling and the small sample size, these preliminary observations warrant further validation in future studies using individual-level sampling and targeted functional experiments.

It is important to acknowledge that there are potential limitations in this study. The small sample size per group (*n* = 5) and pooled intestinal content sampling strategy for omics analysis limit the statistical power of our analyses, preclude assessment of individual biological variation, and restrict the biological interpretation of the observed microbial and metabolic differences. This study explores correlative patterns between growth phenotypes and gut microbiome-metabolome features in Oplegnathus punctatus. The identified microbial and metabolic biomarkers require validation in larger, individually-sampled cohorts across diverse biological contexts. Future studies with increased sample sizes and individual-level multi-omics analyses are needed to validate these preliminary findings, assess inter-individual biological heterogeneity, and perform targeted functional validation experiments. This work provides a preliminary reference for subsequent validation research on the links between the gut microbiome-metabolome and growth variation in farmed *Oplegnathus punctatus*.

## Conclusion

This study integrated 16 S rRNA gene sequencing and untargeted metabolomics to characterize correlative associations between gut microbiome composition, intestinal metabolite profiles, and growth performance divergence in *O. punctatus* cultured in RAS. The FGC exhibited a gut microbiome dominated by *Photobacterium* and *Cetobacterium*, with metabolic profiles significantly enriched in pathways related to protein digestion and absorption, ABC transporters, and aminoacyl-tRNA biosynthesis. Conversely, the SGC exhibited higher gut microbial diversity, characterized by the enrichment of Lactobacillus, Weissella, and Pediococcus. Owing to the small sample size and pooled sampling design, all findings represent preliminary correlative observations, and cannot be interpreted as functional or causal relationships. These results provide preliminary insights into the gut microbiome-metabolome association patterns linked to growth performance in farmed *O. punctatus*, and highlight the importance of accounting for host-specific traits and the aquaculture environment when interpreting microbiome-related growth associations. Future studies are required to validate the potential biological roles of core bacterial taxa in larger, individually-sampled cohorts, and to explore whether these correlative patterns can inform targeted microbiome regulation strategies to advance the sustainable and precise development of *O. punctatus* aquaculture.

## Data Availability

The data presented in the study are deposited in the National Center for Biotechnology Information (NCBI) repository, accession number PRJNA1423140（https://www.ncbi.nlm.nih.gov/sra/PRJNA1423140）. The data set supporting the conclusions of this article is included within the article. Data and materials can also be requested from the corresponding author.
